# Mepolizumab in Severe Pediatric Asthma: Certainties and Doubts through a Single-Center Experience and Review of the Literature

**DOI:** 10.3390/children11080895

**Published:** 2024-07-25

**Authors:** Marco Maglione, Melissa Borrelli, Alessandro Dorato, Chiara Cimbalo, Luigi Antonio del Giudice, Francesca Santamaria

**Affiliations:** 1 Department of Translational Medical Sciences, Federico II University, 80131 Naples, Italy; m.maglione@santobonopausilipon.it (M.M.); melissa.borrelli@unina.it (M.B.); alessandro.dorato@unina.it (A.D.); chiara.cimbalo@unina.it (C.C.); luigiantonio.delgiudice@unina.it (L.A.d.G.); 2 Pediatric Emergency Unit, “Santobono-Pausilipon” Children’s Hospital, 80129 Naples, Italy

**Keywords:** mepolizumab, severe asthma, children, adolescents

## Abstract

Background: Although, in most children with asthma, good symptom control is achieved with a low to moderate dose of inhaled corticosteroids, a small group of patients still experiences frequent symptoms, and even severe exacerbations, impairment of lung function, and reduced quality of life. Some of these subjects with severe asthma require biologic drugs as add-on therapy. In the past decade, numerous monoclonal antibodies have been approved for children or adolescents with severe asthma, in addition to their increasing use in adult asthma. However, the available evidence on how to select the most appropriate biologic based on a single patient’s clinical, functional, and laboratory characteristics is still scant, and is insufficient to guide clinicians in the decision-making process of a personalized treatment. Materials and Methods: We report a case series of four patients with severe eosinophilic asthma treated with mepolizumab, an anti-interleukin-5 monoclonal antibody, and review the existing literature on this treatment in children and adolescents. Results: Our patients, all with blood eosinophilia and elevated fractional exhaled nitric oxide levels, developed poor symptom control despite prolonged treatment with high-dose inhaled corticosteroids *plus* a second controller, addressing the addition of a biologic drug. In all of them, a 12-month treatment with subcutaneous mepolizumab showed a reduction in the blood eosinophil count and in asthma exacerbations, as well as an improvement on the Asthma Control Test. The results of the literature search focused on the strengths and limitations of the pediatric use of mepolizumab and highlighted the areas worthy of further research. Conclusions: Mepolizumab has proven effective in improving symptom control in pediatric patients with severe asthma. Additional well-powered clinical trials will be helpful in developing evidence-based guidelines regarding biologic drugs in the pediatric population.

## 1. Introduction

Chronic asthma affects approximately 10% of children and adolescents [1]. While most patients achieve optimal control with inhaled corticosteroids (ICS) [2], a subgroup ranging from 2% to 10% of asthmatic children may progressively develop the phenotype of severe asthma (SA), characterized by recurrent exacerbations and a continuous need for reliever medications [3].

At all ages, the goal of asthma management is to minimize the disease burden by achieving the best symptom control for the patient [2]. Since their introduction into the therapeutic scenario of SA, biologics have represented both an incredible opportunity and a challenge for children and adolescents [4]. Furthermore, and even more importantly, the development of different monoclonal antibodies has translated the paradigm of personalized medicine into clinical practice, allowing for the possibility to select different molecular targets according to the patient’s endotype [2]. On the other hand, this revolution has taken place over a handful of years, probably too short a time to allow for the production of sufficient scientific evidence to reliably guide clinicians in decision making, particularly in pediatric patients [5].

Currently, biologics for treating pediatric SA include omalizumab, mepolizumab, and dupilumab, which are authorized both in Europe and the US for ages ≥6 years, and benralizumab and tezepelumab, which have been approved in the US and in Europe in children older than 6 and 12 years, respectively.

Despite all biologics belonging to a group of drugs targeting one condition (i.e., severe asthma) [2], if taken individually, they are, in fact, different in many aspects. Molecular targets, clinical effects, and, most of all, the asthma phenotypes in which any beneficial effect has been observed may change when moving from one biologic to another. Pediatric literature indicates that, for all biologics, some evidence supports a reduction in the exacerbations rate and an improvement in asthma control, lung function, or quality of life (QoL) [5]. However, the relevance of these effects may vary depending on the biologic considered. Moreover, the patients enrolled in the available trials significantly differ in terms of age, asthma severity, atopy, lung function, eosinophil count, and exacerbation history, which are all key determinants of treatment efficacy. Hence, a comparison between the efficacy and safety of different biologics might be of little relevance and not recommended [6].

So far, due to the lower prevalence of SA in childhood compared to adulthood, few pediatric studies have adequately addressed issues such as how to choose the most appropriate drug among the several biologics available, how to co-manage them with other antiasthma medications, and how safe they are in the medium- and long-term. Mepolizumab, an anti-interleukin (IL)-5 monoclonal antibody used as add-on therapy in eosinophilic SA, was licensed in 2015 in subjects ≥12 years and in 2019 in those >6 years [7].

Moving from the description of a single-center experience of a subgroup of pediatric SA patients, we explored the evidence provided by a keyword-based search for English articles published on mepolizumab in children and adolescents. We focused on the strengths and limitations of this biologic, highlighting the areas worthy of further research in pediatric SA.

## 2. Materials and Methods

This is a retrospective case series of 4 pediatric patients with severe eosinophilic asthma and symptoms not responding to long-term antiasthma maintenance treatment (medium/high-dose ICS ± other controllers; GINA step 5 [2]) despite the fact that all reversible factors, including allergen exposure, poor treatment adherence, and inhalation technique, had been adequately addressed. For each patient, we described the clinical manifestations of SA, treatment at referral to our unit before therapy with mepolizumab was started, and the course of the disease, along with the main laboratory findings at baseline and after 12 months of mepolizumab. All patients provided written informed consent for the publication of their anonymized clinical and laboratory data.

We also carried out an electronic keyword-based literature search for original articles in English and/or case series published on this topic since January 2004 up to 30 June 2024 in the Scopus, Web of Science, PubMed, and MEDLINE databases. The terms “mepolizumab” AND “children” OR “adolescents” OR “pediatric” OR “severe asthma” were used as keywords in combination. Studies conducted exclusively on adults and anecdotal single case reports were excluded. The identified studies were further evaluated to select relevant literature, and, in addition, a manual search was conducted to evaluate references from review articles.

## 3. Results

### 3.1. Case Series

The charts of 4 children and adolescents (2 boys, 2 girls) with SA, followed at the Pediatric Pulmonology Unit, Department of Translational Medical Sciences, Federico II University, Naples, were reviewed. They were all living in Campania (Southern Italy). Table 1 summarizes the clinical characteristics of the patients at the time of referral to our unit.

Data on lung function, blood eosinophil count, and asthma control at baseline and after 12 months of mepolizumab therapy are reported in Table 2.

We herein briefly describe the individual clinical course before and after treatment with mepolizumab.

*Case 1*

A 14-year-old girl was referred to our center due to inadequately controlled asthma symptoms. Since the age of 3 years, she had experienced frequent and severe asthma exacerbations triggered by both infections and exposure to multiple aeroallergens she was sensitized to. Despite good adherence to the maintenance therapy with inhaled budesonide/formoterol association starting at age 12, symptom control was suboptimal and further reduced by the development of obesity (body mass index (BMI), 31.5 kg/m^2^) and insulin resistance, requiring metformin 2 gr/d and a low-calorie diet. In 2021, a SARS-CoV-2-associated interstitial pneumonia was diagnosed, and hospital admission with intravenous antibiotics and oxygen supplementation was necessary. She recovered well, but in the following year, asthma symptoms and monthly exacerbations, especially in winter and fall, persisted, and due to the presence of severe peripheral eosinophilia, subcutaneous mepolizumab 100 mg monthly was started. Patient-reported symptom control significantly improved soon after the first administrations, with no side effects. Asthma exacerbations significantly decreased in severity and frequency and neither hospital admission nor systemic steroids were required. Inhaled maintenance therapy, which had previously been ineffective, was not modified.

*Case 2*

A 16-year-old girl sensitized to multiple aeroallergens was referred to our unit because of chronic rhinosinusitis with bilateral nasal polyps and monthly asthma attacks. All underlying conditions possibly explaining the clinical picture were ruled out by an extensive diagnostic work-up. One year prior to referral to our unit, she underwent surgical excision of the nasal polyps, but nasal obstruction persisted. Adjustments of asthma maintenance treatment with a step-up strategy, including inhaled budesonide/formoterol and montelukast and repeated assessments of adequate inhaler technique and treatment adherence, did not lead to relevant improvement in asthma control. Her major complaints were poor exercise tolerance and daily chest tightness, which required frequent administration of reliever medications. Given the association with marked blood eosinophilia, monthly administration of mepolizumab (100 mg) was started with only a partial initial response and no change in reported daily symptoms. Nevertheless, at 8 months of treatment, a significant improvement in respiratory symptoms was reported, with particular benefit regarding daily chest tightness and cough. Asthma exacerbations significantly decreased in frequency and severity, and maintenance therapy was stepped down with discontinuation of montelukast, but not of the inhaled budesonide/formoterol association. Systemic steroids were not prescribed, and no hospital admission was required.

*Case 3*

Patient 3 was a 10-year-old boy with multiple perennial inhalant and food allergen sensitization and frequent severe asthma exacerbations since the age of 8 months. We promptly excluded cow’s milk and eggs from the diet. The diet was strictly followed. Because, in the first 7 years of life, four episodes of pneumonia occurred, a thorough diagnostic work-up to exclude underlying conditions was carried out, and no genetic respiratory disease or immune defect was detected. His parents reported approximately one asthma exacerbation per month since age 7, with two more severe attacks requiring hospital admission and oxygen supplementation. Progressive step-up of maintenance therapy with inhaled budesonide/formoterol association and montelukast did not result in any relevant clinical benefit despite good adherence to treatment. Monthly subcutaneous mepolizumab (40 mg/dose) was started at age 10. After 12 months of therapy, the frequency of exacerbations dropped to one every 3–4 months, with a progressively decreased used of systemic steroids and reliever medications. One year after the introduction of mepolizumab, skin prick tests for egg yolk and white were negative (wheal size: 0 mm). Therefore, the patient underwent a challenge with cooked eggs, and, given the negative response, a diet also including egg and its derivatives was prescribed. As far as cow’s milk proteins, a persistent large size of the wheal (>10 mm) as a result of a alfa-lactalbumin, beta-lactoglobulin, and casein skin prick test contraindicated the introduction of cow’s milk into the diet. At present, the patient is in treatment with inhaled budesonide/formoterol association plus montelukast, and no hospital admissions have been notified. The diet still excludes cow’s milk, but includes egg and its derivatives.

*Case 4*

This 14-year-old boy had a history of recurrent lower respiratory infections starting at the age of 2 months, which often required hospital admission and oxygen supplementation. When he was 10 years old, SA was diagnosed, with sensitization to multiple aeroallergens and poor symptom control despite adequate adherence to maintenance antiasthma treatment with ICS *plus* long-acting beta-2 agonists (LABA) and montelukast. The clinical picture was further complicated by obesity (BMI, 29.8 kg/m^2^) associated with insulin resistance, warranting metformin treatment (2.5 gr/d) and a low-calorie diet. A wide diagnostic work-up excluded underlying conditions possibly explaining the clinical picture, e.g. immune defects, primary ciliary dyskinesia, and cystic fibrosis. Daily maintenance therapy with inhaled beclomethasone (200 µg/d) *plus* formoterol was started, with persistent poor symptom control. Treatment with subcutaneous omalizumab was decided at the age of 12 years, but unsatisfactory control persisted and no reduction in asthma exacerbations was observed, with a frequent need for oral steroids. After 24 months of treatment with omalizumab, the patient was referred to our unit. Given the consistently high blood eosinophil count, we decided to discontinue omalizumab, and the patient was switched from omalizumab to monthly subcutaneous mepolizumab (100 mg) after 1 month of washout. A significant decrease in the previously reported frequency of twice-monthly asthma exacerbations, no hospital admissions, and a reduced need for systemic steroids were observed. Maintenance of antiasthma treatment with inhaled budesonide/formoterol association was prescribed.

### 3.2. Review of the Literature

Table 3 summarizes the main findings from nine original articles reporting data on pediatric patients with SA, including subjects aged either >6 years or >12 years. Of all studies (six randomized, double-blind, controlled, multicenter trials [8,9,10,11,12,13]; one non-randomized, open-label study [14]; one real-life, single-center study [15]; and one retrospective single center trial [16]), the oldest was dated 2012 [13] and the most recent were published in 2024 [8,16]. The inclusion criteria for patients were increased sputum or blood eosinophil count plus deterioration of asthma control after a reduction in maintenance ICS or oral corticosteroids (OCS), and/or despite daily treatment with high ICS dose. Of all studies, six also included in the criteria for patient’s enrollment >2 asthma exacerbations treated with systemic steroids in the previous 12 months [8,9,10,12,14,16]. The duration of treatment with mepolizumab ranged from 12 to 52 weeks, and the preferred route of administration was subcutaneous (8/9 studies). The main findings were an improvement of asthma control with fewer asthma exacerbations or hospitalizations and decreased daily OCS dose, or better health-related QoL. Lung function parameters, assessed in eight of the nine studies, were found to be improved in four studies [8,10,12,15].

## 4. Discussion

Biologic therapies, including anti-immunoglobulin E (anti-IgE) (omalizumab), anti-IL-4Rα (dupilumab), anti-thymic stromal lymphopoietin (anti-TSLP) (tezepelumab), and anti-IL-5 drugs (mepolizumab, reslizumab, and benralizumab), play a pivotal role in the management of eosinophilic asthma [17].

The use of mepolizumab, an anti-IL-5 monoclonal antibody that blocks the linkage of IL-5 produced by TH2 and T2 innate lymphoid cells to cell receptors, results in lower production and survival of eosinophils. Several randomized clinical trials (RCTs) in adults and adolescents with eosinophilic SA have contributed to building robust evidence regarding the efficacy of mepolizumab in decreasing the risk of exacerbations and the related need for OCS, as well as improving asthma control and QoL [10,12,13]. Nonetheless, most subjects included in the trials were older than 18 years, thus making their conclusions poorly extendible to younger children [18]. At present, data on mepolizumab in both adolescents and younger children are far more limited. The MUPPITS-2 multicenter RCT of 290 patients aged 6–17 years showed a significant reduction in the number of asthma exacerbations in patients treated for 52 weeks with mepolizumab versus those receiving a placebo [0.96 (95% CI 0.78–1.17) and 1.30 (1.08–1.57), respectively, *p* = 0.027] [9]. Treatment was generally tolerated well, except for higher rates of injection-site reactions associated with mepolizumab than with the placebo. Smaller, non-randomized open-label studies have confirmed a positive clinical profile and no relevant long-term safety issues in children aged 6 to 11 years [14,19]. These findings have been further supported by studies from single pediatric centers on fewer patients, confirming that mepolizumab is effective in reducing asthma exacerbations in children as well in adolescents with SA [15,16,20].

Despite the overall agreement on the positive risk–benefit profile of mepolizumab in children with SA, some criticisms deriving from the comparison between pediatric and adult studies deserve to be mentioned [18]. Indeed, in contrast with what was observed in adults in the DREAM study [13], the reduction in asthma exacerbations found in the pediatric MUPPITS-2 RCT was much less striking [9], thus raising doubts regarding a possible patient selection bias of this trial or, alternatively, on a different efficacy of mepolizumab in different age groups. Particularly, Jackson and coworkers included children with relatively mild blood eosinophilia (150 cells/μL) [9], whereas other large trials in adults [10,13], and even smaller pediatric studies, considered a higher threshold (300 cells/µL, at least once in the previous year) [14,16]. Therefore, as the blood eosinophil count is higher in children than adults [21] and represents only a surrogate measure of airway eosinophilia, the appropriateness of this selection criterion may be questioned, and its impact on the observed results may not be excluded. Furthermore, as acknowledged by the authors, the subjects enrolled in the MUPPITS-2 study were disadvantaged urban Black or Hispanic children, who face several social and environmental health determinants, resulting in a raised burden of morbidity from asthma [9]. The impact of the exposure to pollution and tobacco on respiratory symptoms, or even the occurrence of non-eosinophilic inflammation due to recurrent viral infections, should be considered for future trials of SA children, as a negative effect of these factors on treatment response cannot be neglected. On the other hand, a possible variation in the effect of mepolizumab with increasing age has been hypothesized [22]. Despite the fact that many details concerning the pathophysiology of pediatric SA are still lacking, this consideration is supported by the observation that IL-5 levels in bronchoalveolar lavage (BAL), which indeed correlate with both BAL and blood eosinophil count, increase with age [23]. Finally, some of the patients enrolled in the MUPPITS-2 study showed persistent mucus overproduction associated with eosinophil activation, thus suggesting that refractory mechanisms of eosinophils and epithelium-regulating mucins may occur in SA and thus contribute to an incomplete response to mepolizumab in children and adolescents [9].

Regardless of their impact on asthma control and QoL, a relevant field of comparison between the several biologics available is represented by their effect on asthma-related airway remodeling, which is a crucial target in asthma treatment and a major research area [24]. The strongest evidence of an impact on airway remodeling, also due to the largest experience of its clinical use, is available for omalizumab. Despite the lack of pediatric data, several adult studies analyzing bronchial biopsies [25], matrix metalloproteinases from BAL [26], and airway wall thickness upon chest computed tomography (CT) [27,28,29] support the efficacy of omalizumab in preventing and improving airway remodeling. The effects of mepolizumab on airway remodeling have been less extensively investigated. A study of bronchial biopsies from mild asthmatic adults compared to controls showed a significantly decreased expression of extracellular matrix proteins after three doses of mepolizumab [30]. The CT-measured airway wall thickness and total wall area appeared to be significantly reduced in subjects treated with mepolizumab as compared with those receiving a placebo [31]. Finally, a study analyzing the serum proteomic profiles from 18 adult patients with eosinophilic SA at 1 and 6 months of treatment with mepolizumab compared to healthy controls postulated an impact of mepolizumab on proteins involved in blood coagulation, cell adhesion, and extracellular matrix rearrangement, strongly supporting an effect of mepolizumab on airway remodeling [32]. Unfortunately, all of the above studies were conducted in adults with SA, and no studies have demonstrated a suppressive effect on airway remodeling in childhood SA [33]. Further research, possibly including children, is strongly needed to clarify whether mepolizumab may impact medium- and long-term structural airway modifications of SA. Ultimately, the economic burden associated with the increasingly wide application of drugs as expensive as biologics cannot be ignored. Several studies on children given a biologic drug, particularly omalizumab, have shown significant reductions in the mean consumption of ICS/LABA and short-acting β2 agonists, suggesting that better asthma control and fewer asthma attacks also reduce healthcare-related costs [34,35,36]. Cost-effectiveness data on treatment with mepolizumab are controversial. Treating adults with mepolizumab is not considered cost-effective in the light of the threshold of some countries [37,38,39], but the Severe Asthma Network Italy found that a 12-month treatment of one adult with mepolizumab resulted in a relative cost reduction of −61.8% after excluding the price of the mepolizumab [40]. Pediatric reports on the economic effects of mepolizumab treatment are scarce, with only one US study of insurance claims showing no significant change in asthma medications dispensed in children and adolescents [41].

The reported cases highlight some aspects of the SA process that may improve patients’ care. Our patients experienced their first asthma symptoms at a variable age (from 2 months to 12 years); were sensitized to multiple allergens; and, when referred to our Unit, exhibited blood eosinophilia and elevated FeNO. All had developed SA as defined by ERS/ATS guidelines, i.e., asthma requiring prolonged treatment with high-dose ICS plus a second controller and/or systemic steroids to prevent it from becoming uncontrolled, or asthma that remained uncontrolled despite this therapy [3]. The current SA program at our unit includes the periodic assessment of the inhalation technique as well as a multidisciplinary approach to comorbidities. In all subjects, inhaler skills, treatment adherence, and second- and third-hand smoke exposure were excluded as causes of inadequate control. Two cases were obesity and insulin resistance, and one patient had bilateral nasal polyposis (requiring polypectomy), but poor control of asthma persisted even after comorbidities were treated. Even though the exact role played by comorbidities in pediatric asthma control is not fully understood, they should be sought and treated appropriately [42,43]. Persistence of symptoms with frequent asthma attacks requiring additional systemic steroids despite optimal therapeutic adherence, as well as treatment of comorbidities, indicates the need for additional drugs [2]. In our patients, one-year treatment with mepolizumab was clinically effective in reducing asthma exacerbations by more than 50%. The improved control was also confirmed by an increase in the ACT score, which is a valuable and reliable outcome parameter of response to treatment [8,9,10,11,12,13,14,16,19]. Laboratory findings indicated that post-treatment blood eosinophils were consistently reduced in number (81% to 93% decrease), as shown by previous studies [8,9,11,12,13,14,16]. Spirometry, on the other hand, showed no substantial changes in post-treatment FEV_1_ or FEF_25–75_ compared to pre-treatment values. Although findings from a small case series are not generalizable to a broader population, it is worth mentioning that data on the lung function effects of mepolizumab are controversial [43]. Adults’ studies have concluded that the best effect of mepolizumab on spirometry is achieved in subjects with higher baseline sputum and/or blood eosinophils, which may indeed indicate that the stronger the eosinophilic inflammation, the better the lung function improvement [44]. Of all pediatric studies also assessing spirometry, 50% found a significant improvement of FEV_1_ [8,10,12,15], and others did not [11,13,14,16]. 

With regard to QoL, despite not being specifically assessed in the present case series, an impact of treatment on patients’ reported outcomes is highly likely, in similarity to what has been observed by several trials of mepolizumab in adolescents and adults [8,10,12]. For other biologics, namely dupilumab, the timing of QoL improvement has been addressed in detail and a quick effect within 4 weeks after treatment onset has been documented [45]. Such information was not reported in trials assessing QoL changes after mepolizumab, but, in our experience, improvements are reported within 1–2 months after the first administration, even though we observed significant individual variability. Among the benefits often reported by patients, the reduction in use of asthma medications is of main relevance. Indeed, the use of fewer drugs combined with the perception of better symptom control tends to increase patients’ compliance, which is a crucial issue, particularly in young adolescents. Finally, in this case series, post-treatment FeNO levels were reduced in two and remained unmodified in two out of four cases, respectively. The literature on mepolizumab’s effects on FeNO in children is inconclusive, even though significant reduction can be observed in patients with the highest pretreatment FeNO [46]. More pediatric studies are needed, possibly comparing actively treated versus placebo-group subjects, to verify the effects of mepolizumab on lung function and FeNO. 

Final remarks regarding the choice of one biologic rather than another derive from the observation that, in case 4, previous treatment with omalizumab failed to result in better asthma control, while one year-mepolizumab resulted in a reduction in blood eosinophil count, increased ACT, and improved spirometry. Current pediatric literature recommends selecting a biologic based on the patient’s age and biomarkers, such as the allergic asthma indicators blood eosinophil count and FENO levels, or lung function [4]. Despite these important recommendations, many aspects still need to be clarified in the mechanisms that affect biologics’ efficacy on clinical outcomes, and recent evidence suggests that, for instance, specific eosinophil subpopulations may not decrease with mepolizumab treatment, showing an association with persistent exacerbations [47]. On the other hand, transcriptomic analyses allowed us to identify the inflammatory patterns that contributed to exacerbations despite reducing eosinophil-related inflammation. Higher baseline expression of these pathways involving the eosinophil, inclusive of canonical T2 inflammation and eicosanoid metabolism, has been associated with a better response to mepolizumab [9]. Such findings further contribute to extending the areas worthy of further research regarding the application of mepolizumab and other biologics in pediatric SA, increasing the number of unanswered questions. These include how to choose when treatment should be started, which are the most appropriate tools for assessing patients’ responses, how to co-manage biologics and standard asthma therapy, when to consider stopping or switching to other options, and, more importantly, whether these drugs may affect the natural history of asthma [5,48].

Although limited, this case series and the examination of the available literature on pediatric application of mepolizumab leads us to conclude that this biologic drug has proven to be effective in reducing asthma exacerbations in pediatric patients with SA showing high blood eosinophil counts. However, additional well-powered pediatric clinical trials would be helpful in developing evidence-based guidelines regarding biologic therapies in this population.

## Figures and Tables

**Table 1:** Clinical characteristics of the described patients.

	Case 1	Case 2	Case 3	Case 4
Age at asthma onset	3 years	12 years	8 months	2 months
Allergic sensitization	House dust mites, cat dander, *Olea europaea*, *Parietaria judaica*	Grass pollen, Artemisia, *Olea europaea*, *Parietaria judaica*	House dust mites, cat/dog dander, cow’s milk proteins, egg	House dust mites, *Alternaria*, *Olea europaea*, *Parietaria judaica*
Symptom burden	Weekly cough, dyspnea, and night awakenings. Frequent asthma attacks requiring systemic steroids, extra ICS, and bronchodilators.	Daily chest tightness, cough, exercise-induced dyspnea. Frequent asthma attacks requiring systemic steroids, extra ICS, and bronchodilators.	Monthly exacerbations with frequent need for systemic steroids, extra ICS, and bronchodilators.	Exacerbations requiring systemic steroids twice a month. Weekly night awakenings due to respiratory symptoms.
Comorbidity	Obesity, insulin resistance	Nasal polyposis	-	Obesity, insulin resistance
Treatment at referral	Budesonide (640 µg/d) + Formoterol (18 µg/d)	Budesonide (640 µg/d) + Formoterol (18 µg /d) + Montelukast (10 mg)	Budesonide (640 µg /d) + Formoterol (18 µg/d) + Montelukast (10 mg)	Beclomethasone (200 µg/d) + Formoterol (12 µg/d) + Omalizumab (450 mg/14 d)
Age at mepolizumab	14 years	16 years	10 years	14 years

**Table 2:** Summary of spirometry, laboratory, and clinical findings of the patients at baseline and after 12 months of mepolizumab.

	Case 1		Case 2		Case 3		Case 4	
	Baseline	12 Months	Baseline	12 Months	Baseline	12 Months	Baseline	12 Months
Blood eosinophils, cells/µL (%)	1490 (13)	140 (1.3)	1470 (19)	100 (1.4)	420 (8.2)	80 (1.8)	530 (6)	70 (0.8)
Decrease in eosinophil count post-mepo (%)	−91		−93		−81		−86	
FEV_1_, L (% pred)	3.7 (125)	3.1 (89)	3.3 (84)	3.0 (86)	1.5 (94)	1.5 (89)	1.6 (37)	4.3 (99)
FEF_25–75_, L/s (% pred)	2.5 (67)	2.9 (73)	3.7 (84)	2.8 (65)	1.3 (64)	0.9 (44)	0.7 (16)	4.2 (87)
Total serum IgE (IU/mL)	1900	1800	234	250	1980	1980	1560	403
FeNO	143	117	53	55	68	33	38	12
ACT score	12	18	15	20	18	21	8	15
No. of exacerbations/year	18	7	20	9	17	5	18	8
Current treatment	Mepolizumab + Budesonide (640 µg/d) + Formoterol (18 µg/d)		Mepolizumab + Budesonide (640 µg/d) + Formoterol (18 µg/d)		Mepolizumab + Budesonide (640 µg/d) + Formoterol (18 µg/d) + Montelukast (10 mg)		Mepolizumab + Budesonide (640 µg/d) + Formoterol (18 µg/d)	

Abbreviations: FEV_1_: forced expiratory volume in 1 s; FEF_25–75_: forced mid-expiratory flow; FeNO: fractional exhaled nitric oxide; mepo: mepolizumab; ACT: asthma control test, as provided at https://www.asthmacontroltest.com/en-gb/welcome/, accessed from 10 January 2021 to 30 December 2023.

**Table 3:** Main characteristics of pediatric studies assessing the effects of mepolizumab.

Reference	Study Design	Number of Cases and Age	Inclusion Criteria	Mepolizumab Daily Dose/Duration	Main Findings
**Pavord, 2012** **(DREAM) [13]**	Randomized, double-blind, controlled, multicenter trial	616; 12–74 years (156 assigned to mepo 750 mg, 152 to mepo 250 mg, 153 to mepo 75 mg, 155 to placebo)	Sputum EOS > 3%, F_ENO_ > 50 ppb, blood EOS >300 cells/μL, or asthma control deteriorating after <25% reduction in maintenance ICS or OCS. Daily treatment with ≥880 μg inhaled fluticasone or equivalent, with or without maintenance OCS, and additional controller drugs.	750 mg, 250 or 75 mg s.c. every 4 weeks for 48 weeks	Decreased asthma exacerbations (all doses) versus placebo. No FEV_1_ change.
**Ortega, 2014** **(MENSA) [12]**	Randomized, double-blind, double-dummy, controlled, multicenter, phase 3b trial	576; 12–82 years (194 assigned to s.c. mepo 100 mg, 191 to i.v. mepo 75 mg, 191 to placebo)	Blood EOS > 300 cells/μL in the previous year, or >150 cells/μL at screening. >2 exacerbations treated with SCS in the previous year. Daily treatment with ≥880 μg inhaled fluticasone or equivalent and ≥3 months of treatment with an additional controller. FEV_1_ < 80% pred (age ≥18 years) or <90% pred (age 12–17 years).	75 mg or 100 mg s.c. every 4 weeks for 32 weeks	Decreased asthma exacerbations. Improvement of FEV_1_ and QoL with both s.c. and i.v. mepo.
**Bel, 2014** **(SIRIUS) [11]**	Randomized, double-blind, controlled, parallel-group, multicenter trial	135; 16–74 years (69 assigned to s.c. mepo 100 mg, 66 to placebo)	Blood EOS > 300 cells/μL in the previous year, or >150 cells/μL at screening. Maintenance SCS therapy >6 months. Treatment with high-dose ICS + an additional controller.	100 mg s.c. every 4 weeks for 20 weeks	Decreased asthma exacerbations. Steroids-sparing effect. Improved asthma control. Non-significant trend of FEV_1_ improvement.
**Chupp, 2017** **(MUSCA) [10]**	Randomized, double-blind, controlled, parallel-group, multicenter, phase 3b trial	551; ≥12 years (274 assigned to mepo, 277 to placebo)	Blood EOS > 300 cells/μL in the previous year, or >150 cells/μL at screening. >2 exacerbations treated with SCS in the previous year. FEV_1_ <80% pred (age ≥18 years) or <90% pred (age 12–17 years).	100 mg s.c. every 4 weeks for 24 weeks	Improved QoL and FEV_1_.
**Gupta, 2019 [14]**	Non-randomized, open-label, repeat-dose, phase 2 study	36; 6–11 years	Blood EOS ≥150 cells/μL at screening or ≥300 cells/μL in the previous year. ≥2 exacerbations treated with SCS in the previous year. Treatment with >200 μg/d fluticasone propionate or equivalent with/without maintenance OCS and ≥1 controller drug.	40 mg (<40 kg) or 100 mg (>40 kg) s.c. every 4 weeks for 12 weeks	Trend toward improved asthma control. Favorable safety profile No FEV_1_ change.
**Jackson, 2022** **(MUPPITS-2) [9]**	Randomized, double-blind, controlled, parallel-group, multicenter trial	290; 6–17 years (146 assigned to mepo, 144 to placebo)	Blood EOS ≥150 cells/μL. ≥2 exacerbations treated with SCS in the previous year. Twice-daily therapy with at least fluticasone propionate 250 μg or equivalent (6–11 years), or at least futicasone/salmeterol 250/50 μg or equivalent (12–17 years).	40 mg (age 6–11 years) or 100 mg (age 12–17 years) s.c. every 4 weeks for 52 weeks	Decreased asthma exacerbations.
**Wetzke, 2022** **[15]**	Real-life multicenter study	18; 6–17 years	Uncontrolled asthma despite high ICS/LABA doses and trigger avoidance, or asthma requiring high doses of ICS/LABA to remain controlled	40 mg (age 6–11 years) or 100 mg (age 12–17 years) s.c. every 4 weeks for 12.3 (median; range, 3–36) months	No significant reduction in exacerbation rate except 4 cases with follow-up >1 year. Improved FEV_1_.
**Lim, 2024 [16]**	Retrospective, single-center study	16; 7–17 years	Blood EOS >300 cells/μL or F_ENO_ > 50 ppb in the previous year. ≥3 exacerbations treated with SCS in the previous year. Treatment with high-dose ICS + an additional controller.	40 mg (age 6–11 years) or 100 mg (age 12–17 years) s.c. every 4 weeks for 48 weeks	Decreased asthma-related hospitalizations. Reduced OCS dose/day No FEV_1_ or FEF_25–75_ change.
**Chen, 2024 [8]**	Randomized, double-blind, controlled, parallel-group, multicenter trial	300; ≥12 years (149 assigned to mepo, 151 to placebo)	Blood EOS ≥ 150 cells/μL at screening or ≥300 cells/μL in the previous year. ≥2 exacerbations treated with SCS in the previous year. FEV_1_ < 80% pred (age ≥18 years) or <90% pred (age 12–17 years). Treatment with high-dose ICS + an additional controller.	100 mg s.c. every 4 weeks for 52 weeks	Decreased asthma exacerbations and asthma-related hospitalizations. Improved QoL and FEV_1_.

Abbreviations: yrs, years; EOS, eosinophils; F_ENO_, fractional exhaled nitric oxide; ppb, part per billion; QoL, quality of life; ICS, inhaled corticosteroids; OCS, oral corticosteroids; SCS, systemic corticosteroids; mepo, mepolizumab; FEV_1_, forced expiratory volume during the first second; s.c., subcutaneous; i.v., intravenous.

## Data Availability

The data presented in this study are available upon request from the corresponding author. The data are not publicly available due to privacy restriction.
